# Development of a RP-HPLC method for determination of glucose in *Shewanella oneidensis* cultures utilizing 1-phenyl-3-methyl-5-pyrazolone derivatization

**DOI:** 10.1371/journal.pone.0229990

**Published:** 2020-03-12

**Authors:** Norberto M. Gonzalez, Alanah Fitch, John Al-Bazi

**Affiliations:** 1 Department of Chemistry and Biochemistry, Loyola University Chicago, Chicago, IL, United States of America; 2 Department of Chemistry, Northeastern Illinois University, Chicago, IL, United States of America; University of Pisa, ITALY

## Abstract

A method was developed and validated for low-level detection of glucose. The method involves quantitation of glucose though derivitization with 1-phenyl-3-methyl-5-pyrazolone (PMP) and HPLC-DAD analysis. The developed method was found to be accurate and robust achieving detection limits as low as 0.09 nM. The applicability of the method was tested against microbial samples with glucose acting as a carbon fuel source. The method was shown to be able to accurately discriminate and quantify PMP-glucose derivatives within *Shewanella oneidensis* MR-1 samples. The method proved capable at examining glucose usage during the early hours of microbial growth, with detectable usage occurring as early as two hours. *S*. *oneidensis* cultures were found to grow more effectively in the presence of oxygen which coincided with more efficient glucose usage. Glucose usage further increased in the presence of competing electron acceptors. The rate at which *S*. *oneidensis* reached exponential growth was affected by the presence of ferric iron under microaerobic conditions. Such samples reached exponential growth approximately two hours sooner than aerobic samples.

## Introduction

Microorganisms can utilize organic material to facilitate culture growth, which can include simple organic carbons such as acetate, lactate, fumarate and, to a lesser extent, carbohydrates such as glucose [1–3]. Understanding the relative use of these substances may be important in a wide range of fields, one example of which is the use of a carbon source by bacteria for microbial fuel cell development.

Most methods are individual to the carbon source and may involve a wide number of strategies. In the case of glucose common methods include glucose assays [4], copper-iodometric methods [5], mass spectrometry [6], and chromatographic methods to include gas chromatography and high-performance liquid chromatography (HPLC) [7–9]. Glucose assays are among the fastest and simplest forms of analysis. They suffer, however, from high cost with numerous samples and are limited to one or only a few reducing sugars. Furthermore, a survey of glucoses assays available from leading manufacturers showed method sensitivities as low as 1 μM limiting their use for biological detections [10–13].

**Fig 1:**
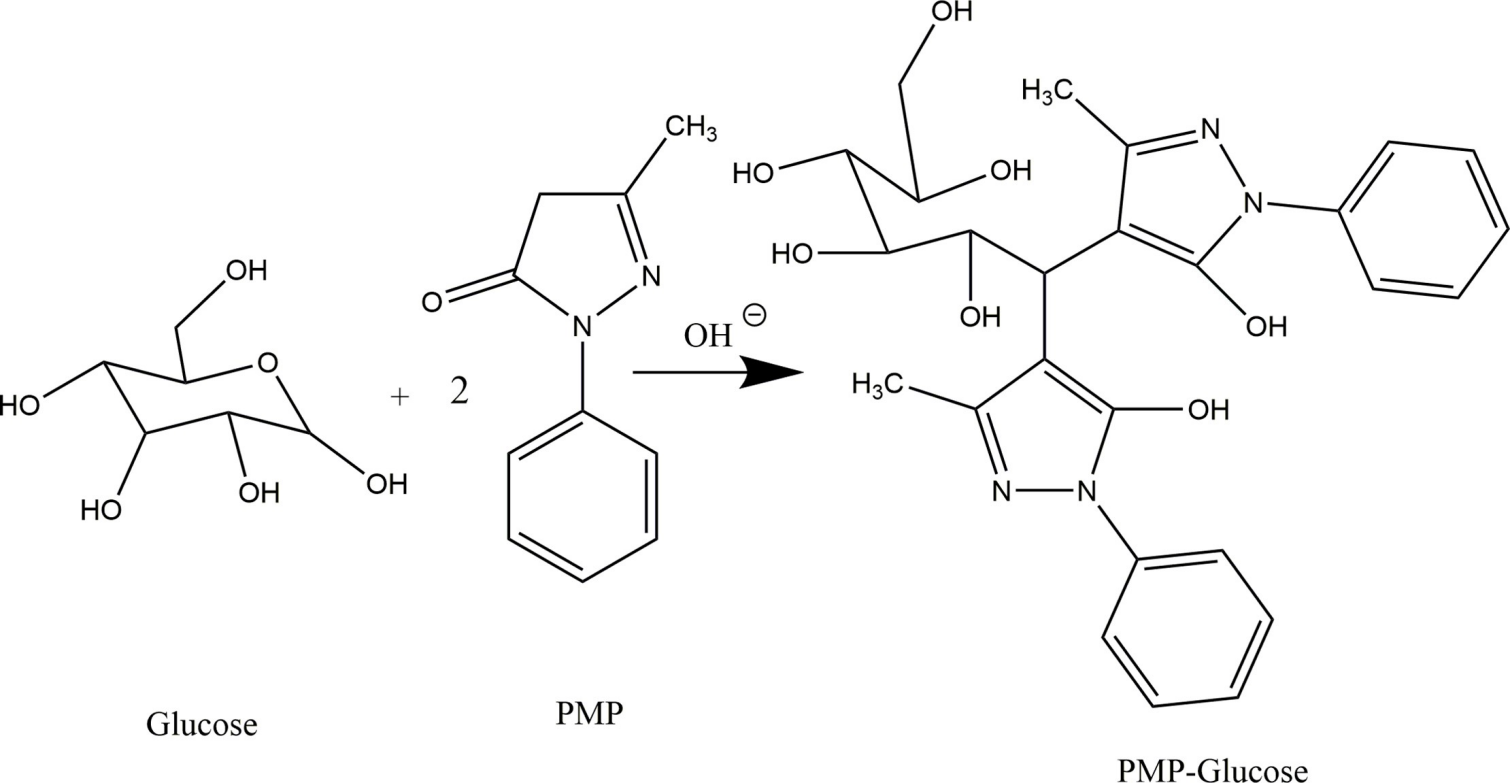
A reaction scheme for the formation of PMP-Glucose. One equivalent of glucose reacts with two equivalents of PMP to form one equivalent of PMP-Glucose under basic conditions.

HPLC methods allow for a decrease in cost when testing numerous samples and for quantitation of various reducing sugars either individually or simultaneously. An increasing popular LC method involves the use of derivatizing reducing sugars like glucose with 1-phenyl-3-methyl-5-pyrazolone (PMP) (Fig 1). First proposed by Honda et al. [14] the method involves directly reacting PMP with a reducing sugar under basic conditions to form a PMP-reducing sugar derivative that can be used for observing multiple reducing sugars simultaneously in the ultraviolet spectrum. While the method has been refined by various research groups [15–17] to include unicellular algae [18], the usefulness of the method for analysis of bacterial cultures has not been examined. Many current methods of PMP derivatization suffer from long retention times exceeding 25 minutes and inconsistent reaction times ranging from 30–120 minutes. Further, many methods suffer from a lack of full validation for LC methods set by such governing agency as the International Council on Harmonisation of Technical Requirements for Pharmaceuticals for Human Use (ICH) [19,20]. Proper method validation would improve method robustness and repeatability.

In this study, a modified PMP method was developed for examining glucose. The method focuses on achieving decreased run times, improved method robustness through method validation, and achieving lower detection limits not previously seen in similar works. The applicability of the method was tested using the microorganism *Shewanella oneidensis* MR-1 and examines the rate of glucose consumption by the microbe over time. Several publications attempted to address the ability of *Shewanella* to utilize glucose utilizing different instrumental determinations of glucose. Work conducted by Nakagawa et al. [21] utilized a genetically altered *Shewanella* strain and enzymatic assays for glucose measurements. Howard et al. [22] also utilized a mutated *Shewanella* strain, however, the group incorporated LC method with refractive index (RI) detection for glucose detection. Likewise, Choi et al. [23] also incorporated the use of LC and RI detection of glucose used by a mutated *Shewanella* strain. Rosenbaum et al. [24] used a wild type from of *Shewanella* and HPLC with pulsed amperometric detection (PAD) for quantifying glucose in growth cultures. Although these works have demonstrated that refractive index detectors can detect glucose in growth media, RI detectors suffer from limited sensitivity and gradient incompatibilities that reduces their ability to achieve low detection limits.

*S*. *oneidensis* was chosen as the model for three reasons. Firstly, the organism, in general, is of intense interest due to its utilization in microbial fuel cells [25–29]. *S*. *oneidensis* is a facultative anaerobe used in microbial fuel cells (MFC), and has been shown to be well adapted at using small carbon compounds as a fuel source [30,31]. The microbe has shown difficulty utilizing larger carbon compounds like glucose primarily due to a lack of key enzymes required for glycolysis [22,32–36]. However, some research has suggested *Shewanella* can utilize glucose when the microbe is allowed to condition in a glucose containing solution, or when the microbe has adapted it metabolism to an oxygenated environment prior to growth [37]. Secondly, the use of PMP derivatization and HPLC analysis has not been previously seen for tracking carbohydrate utilization by *S*. *oneidensis*. Finally, current literature has focused on the utilization of glucose over an extended growth window. Few publications have focused on examining the use of glucose during early (0–15 hours) microbial growth and at which point glucose utilization begins.

## Materials and methods

### Standards and reagents

The mobile phase organic solvent acetonitrile was purchased from Fisher Chemical and was of HPLC grade. Potassium phosphate dibasic was purchased from Sigma-Aldrich and was of ACS grade. The glucose standard was purchased from Fisher Chemical and was of ACS grade. The derivatizing reagent 1-phenyl-3-methyl-5-pyrazolone (PMP) was purchased from Sigma-Aldrich and was of ACS grade. The tryptic soy broth (TSB) growth medium was purchased from Sigma-Aldrich and was of microbiology grade. Iron oxide and iron chloride were purchased from Fisher Chemical and were of reagent grade. The *Shewanella oneidensis* MR-1 microbe were purchased from ATCC as a frozen culture.

### Instrumentation

The method development and validation experiments were performed using an Agilent Technologies 1100 Series HPLC system equipped with a quaternary pump, auto sampler, and a diode array detector. The chromatographic data were obtained using Agilent Technologies ChemStation for LC Rev.A10.02[1757] software. The column used was a YMC-Pack ODS-AQ with a column length of 150 mm, an inside diameter of 4.6 mm and a particle size of 3 microns.

### Preparation of standard solutions and samples

#### Stock solutions

Solutions of PMP were prepared by dissolving 87 mg of PMP in 1 mL of methanol to yield a 0.5 M stock solution of PMP-methanol. Glucose stock solutions were prepared by dissolving 0.125 g of glucose in 50 mL of deionized water to yield a 0.0139 M stock solution of glucose. The appropriate aliquot of 0.0139 M glucose stock was used for all other stock solution preparations. Solutions of TSB were prepared by dissolving 30 g of TSB in 1 L of deionized water. All TSB solutions were autoclaved after preparation. For TSB containing ferric iron, both soluble and insoluble iron was first dissolved in the TSB prior to incubation.

#### Standard solutions

Standards of PMP-glucose were prepared by combining 800 μL of a 0.0139 M solution of glucose with 800 μL each of 0.5 M NaOH and 0.5 M solution of PMP in methanol to yield a solution at a pH of 13. The reaction was carried out at 70°C with agitation every 30 minutes. After derivatization, the solution was neutralized at 4°C with 800 μL 0.5 M HCl. Reaction impurities were extracted using 2 mL of dichloromethane. The solution was mixed vigorously for 30 seconds and centrifuged at 4°C and 5000 rpm for 5 mins. The dichloromethane extraction was repeated twice more for a total of 3 extractions. The upper aqueous layer containing the PMP-glucose was removed for analysis.

#### Sample solutions

The TSB was prepared by autoclaving 80 mL of the growth media. Media designated as microaerobic was purged with nitrogen gas as the media cooled to room temperature. Media designed as aerobic was saturated with oxygen gas for 10 minutes. The samples were prepared by adding 1 mL of a *S*. *Oneidensis* MR-1 containing solution to the 80 mL of autoclaved TSB. The microaerobic flasks were sealed with a septa cap to prevent additional oxygen exposure while aerobic flasks were covered with foil. Samples were placed in an incubator set at a temperature of 30°C and a shake speed of 150 rmp. The samples were set to grow for 15 hours with sampling occurring throughout the growth period.

#### *S*. *oneidensis* samples

The analysis of glucose occurred utilizing a single growth culture. Samples extracted from a single culture over a 15-hour incubation period exhibited greater reproducibility when compared to sampling a new culture for every hour of analysis. Additionally, utilizing a single growth culture allowed for samples to be collected every 15–30 minutes when samples exhibited exponential growth. All results were replicated with a second growth culture. Microbial growth was normalized, and the glucose utilization was averaged.

Microbial samples were inoculated in 80 mL of TSB and allowed to incubate under microaerobic and aerobic conditions for 15 hours at 30°C. At various intervals over the growth period, the growth of the microbe was determined by examining its turbidity at 650 nm, and 1 mL of growth media was collected for analysis by HPLC. Growth media samples were derivatized and examined by HPLC in the same manner as the method development and validation samples. Preparation steps included centrifugation of the samples and extraction of the sample supernatant.

## Results and discussion

### Optimization of chromatographic parameters

#### Wavelength selection

The wavelength used for the detection of PMP-glucose was selected by the analysis of glucose, PMP, and PMP-glucose on an offline UV detector. The λ_max_ for PMP- glucose was selected to be 245 nm. The UV spectrum of PMP-glucose using a DAD was used as further evidence for the location of the PMP-glucose peak in sample chromatograms.

#### Reaction optimization

**Fig 2:**
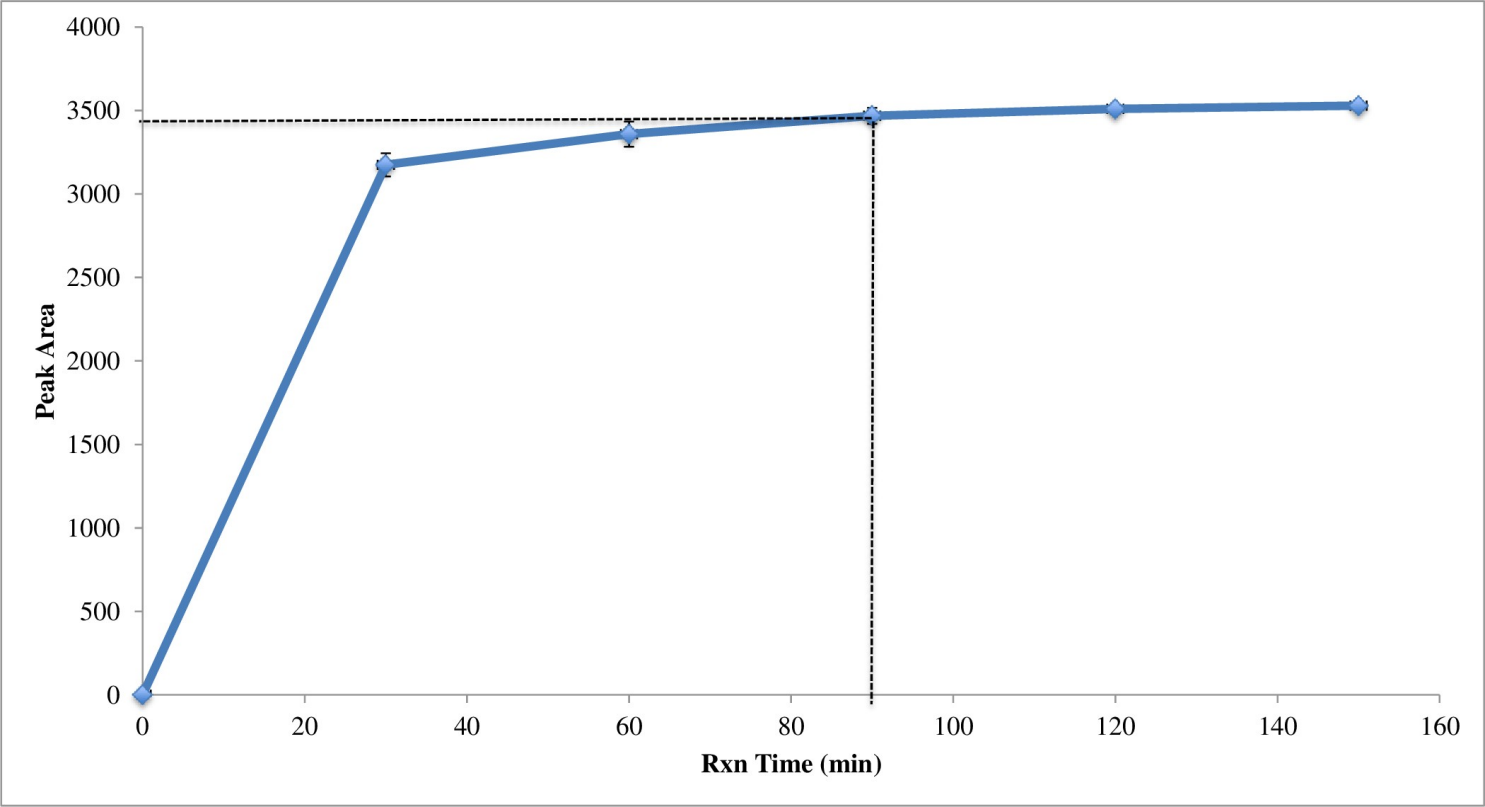
Peak area of PMP-Glucose as a function of derivatization reaction time. The peak area of PMP-Glucose remains constant after 90 minutes indicating that the reaction has reached completion.

The optimal reaction time for the derivatization was performed by multiple reactions ranging from 30 to 150 min. Reaction mixtures were injected and the peak areas were determined and plotted against the reaction time (Fig 2). The optimal reaction time was determined to be 90 minutes, when an increase in the PMP-glucose peak area was no longer observed, indicating that the reaction was complete.

#### Column selection

Both reversed-phase and normal-phase columns were tested for method development using a mobile phase consisting of 15 mM potassium phosphate dibasic pH 7.2 and ACN. Selection of the appropriate column was determined by injecting a sample of PMP-glucose and observing the overall separation and peak characteristics. Based on the analysis of six C_18_, two amino, and two phenyl columns, a YMC-Pack ODS-AQ (150 x 4.6 mm, 3 μm) column was selected which provided a uniform peak with adequate resolution. A 3 μm YMC guard column with a 4.0 mm inside diameter was used to prolong the life of the column.

#### Gradient selection

**Fig 3:**
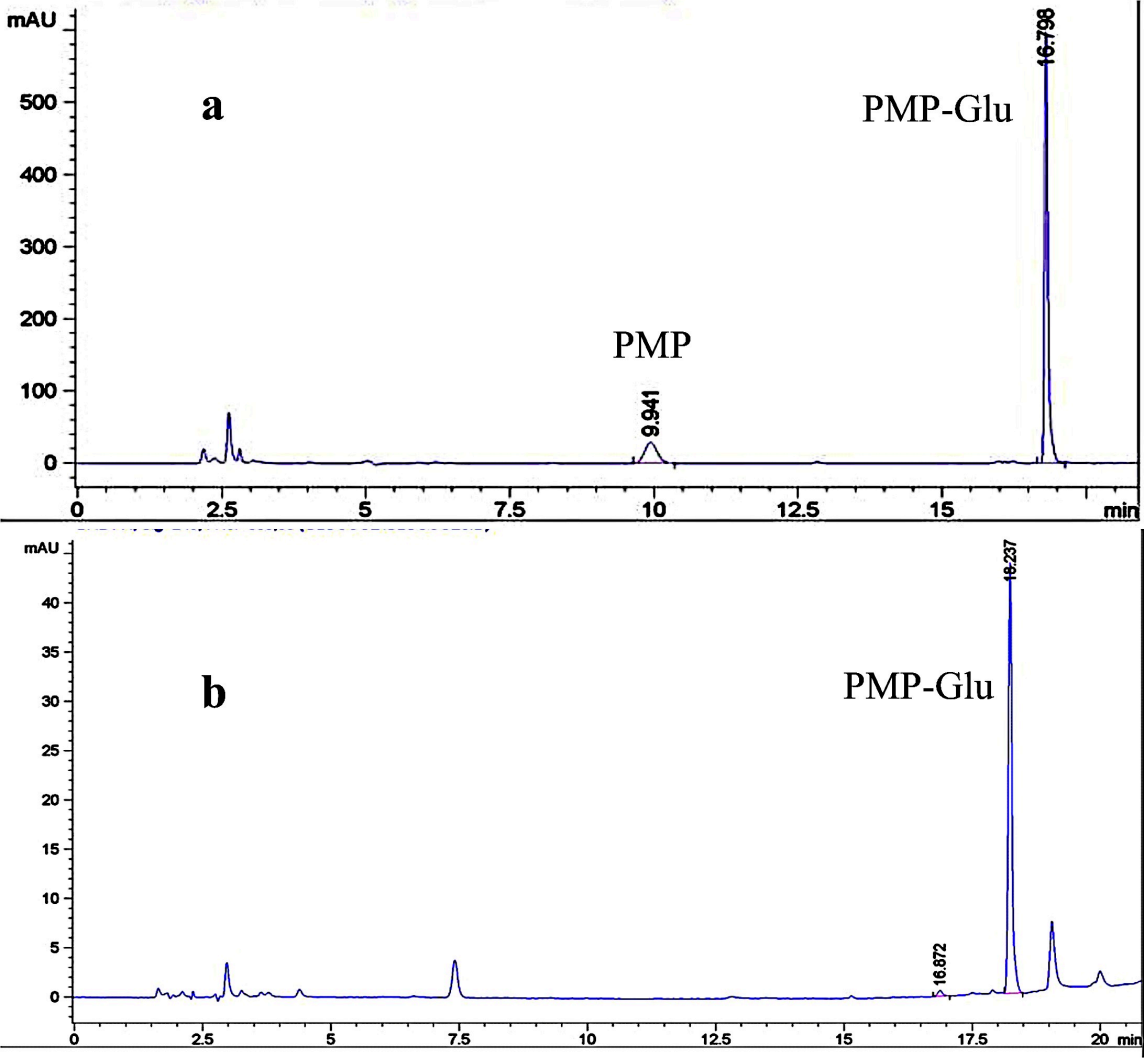
Representative chromatograms for a) blank TSB growth media and b) TSB supernatant extracted from a *S*. *oneidensis* culture.

The separation of PMP-glucose from the corresponding matrix was of interest. This was achieved utilizing a segmented gradient which consisted of 15 mM potassium phosphate dibasic buffer at a pH of 7.2 (Eluent A) and ACN (Eluent B) (Table 1). This gradient provided a reasonable retention time of 16.7 min with significant resolution between the peak of interest and the sample matrix (Fig 3A and 3B).

**Table 1:** Segmented gradient profile.

	Percent composition	
Time (Min)	Eluent A	Eluent B
0	90	10
9	86	14
30	36	64
35	36	64

### Method development

Development of the method focused on maximizing the peak area and theoretical plates while achieving adequate resolution. Optimization of the method parameters examined the effect of varying injection volume, flow rate, temperature, and buffer pH and ionic strength. Based on these studies, the optimal method parameters were selected (Table 2). The method showed a substantial decrease of 10–15 minutes in run time when compared to many other PMP methods.

**Table 2:** Summery of method parameters.

**Column**	YMC Pack ODS-AQ (150 x 4.6 mm, 3 μm)
**Injection Volume**	5 μL
**Mobile Phase**	Eluent A: 15 mM Potassium Phosphate Dibasic pH 7.2 Eluent B: 100% ACN
**Flow Rate**	1 mL/min
**Column Temperature**	29.5°C
**Wavelength**	245 nm
**Run Time**	35 min
**Equilibration Time**	10 min

The decrease in retention time can be attributed to two factors. The column used was 100 mm shorter and had a smaller particle size than those used by other works [15–18]. More importantly, the mobile phase was changed to a phosphate buffer as opposed to the commonly seen acetate buffer. The result is a weaker interaction between PMP-glucose with the stationary phase due to an increase in mobile phase pH. Increasing the mobile phase pH consequently decreased the retention time of PMP-glucose.

### Method validation

#### System suitability

To ensure reliability of the developed method, five injections of an 8.33 mM PMP-glucose sample were performed. The separation parameters were evaluated for each injection to ensure the resolution and reproducibility of the system was adequate (Table 3). Based on the acceptance criteria for each parameter, it was determined that the selected method parameters were acceptable.

**Table 3:** System suitability study.

Parameter	Acceptance Criteria	Mean Values (n = 5)
Plate Number	≥ 2000	399586 ± 39.7
Tailing Factor (T_f_)	0.9 ≥ T_f_ ≤ 2	1.242 ± 0.00242
Resolution	≥ 1.5	7.213 ± 0.0004
% RSD Retention Time	≤ 2%	0.0299 ± 0.0005
% RSD Peak Area	≤ 2%	1.97 ± 0.0835
% Drift Peak Area	≤ 2%	1.09 ± 0.0604

#### Specificity

Specificity studies were performed to ensure no co-eluting impurities with the peak of PMP-glucose. Forced degradation was performed as part of the peak purity studies [19,20,38]. Samples of 1 mL PMP-glucose were subjected to acid and base degradation using 1 mL of various concentrations of HCl and NaOH. The degraded solutions were neutralized with the appropriate acid or base. Oxidation degradation was assessed using l mL of various percentages of hydrogen peroxide. Additionally, l mL of PMP-glucose was subjected to heat and UV degradation. The percent degradation for PMP-glucose was assessed utilizing HPLC. The results showed the greatest degradation occurring under both 3 M NaOH and HCl as well as 0.001% hydrogen peroxide.

The determination of peak purity was conducted by combining 100 μL of the samples subjected to 0.001% H_2_O_2_, 3 M NaOH, and 3 M HCl degradation and injecting this mixture into the HPLC system. Successive scans of the PMP-glucose peak were conducted and overlaid to determine the purity. From these scans, PMP-glucose peak was determined to have a purity of 99.9%. This value is well within the acceptance criteria of 99%. These results indicate that impurities within the reaction mixture will not interfere with the PMP-glucose peak.

#### Robustness

The robustness of the method was tested to measure its capacity to remain unaffected by small variations in the method parameters. Robustness was particularly focused on the resolution of PMP-glucose from adjacent peaks. Small variations in the method parameters were studied by deliberately changing the wavelength, flow rate, buffer pH and ionic strength, and the temperature of the column. Acceptance criteria for method robustness have not been set by the ICH, therefore a lower specification limit (LSL) of 1.5 (baseline resolution) was established as the acceptance criteria. After testing each condition in triplicates, the study showed the method was unaffected by small variations in method parameters and therefore is considered robust (S1 Table).

#### Limit of quantitation and detection

The limit of quantitation (LOQ) and the limit of detection (LOD) for the method were determined by measuring the magnitude of the PMP-glucose peak height to the magnitude of the baseline noise (S/N). A S/N ratio of 10 is acceptable for the LOQ and 3 for the LOD. The LOQ was found to be 0.2 nM with a S/N ratio of 9.8, and the LOD was found to be 0.09 nM with a S/N ratio of 3.7. The values for LOQ and LOD were performed in triplicates. The limit of detection of was found to be an order of magnitude lower than the lowest known LOD for glucose utilizing similar instrumentation [39]. The lower detection limit can be attributed to the use of a column with a particle size of 3 microns when compared to the 5 microns used for other works. Reducing the particle size resulted in an increase in theoretical plates and sharper PMP-glucose peak.

#### Linearity and range

Linearity studies were conducted to measure how well a calibration plot of peak area versus concentration approximates a straight line. Several glucose concentrations covering the range of 16.65 mM to 0.2 nM (the LOQ) were tested. The expected maximum glucose concentration within a sample was 13.87 mM (2.5 g/L). The upper range corresponds to 120% of the maximum glucose concentration within TSB while the lower limit was set to the limit of quantitation, 0.2 nM. A linear regression equation was then processed along with a correlation coefficient (S1 Fig). The correlation coefficient of 0.999 meets the acceptance criteria of ≥ 0.999. The calculated percent bias was determined to be 0.65% meeting the ICH guideline of NMT ± 3% indicating the results are not forced though the origin.

#### Accuracy

The accuracy of the method was determined by investigating how closely the test results reflect the true value. The accuracy was conducted by preparing 3 samples of PMP-glucose in the range of 0.5 to 2.5 g/L. All 3 samples were performed in triplicates. Based on the linear regression equation, the recovered concentrations were calculated. The percent recovery fell within the acceptance range of 98–102% (S2 Table).

#### Precision

The injection precision for the method was tested to measure how similar the results are with repeated injections. A 2.5 g/L sample of PMP-glucose was prepared and injected six times. After averaging the peak areas, a percent RSD of 0.093% was calculated. This value is within the acceptance criteria range of a %RSD ≤ 1.5% (S3 Table).

The method precision was conducted to measure how similar the results are among separate stock solutions. Six samples of PMP-glucose were prepared from separate stock solutions at a concentration of 2.5 g/L and diluted to 1.5 g/L. After injecting each sample, the peak areas were average together and a percent relative standard deviation of 0.16% was calculated (S3 Table). This value meets the acceptance criteria of a percent RSD ≤ 1.5%.

### Method application

**Fig 4:**
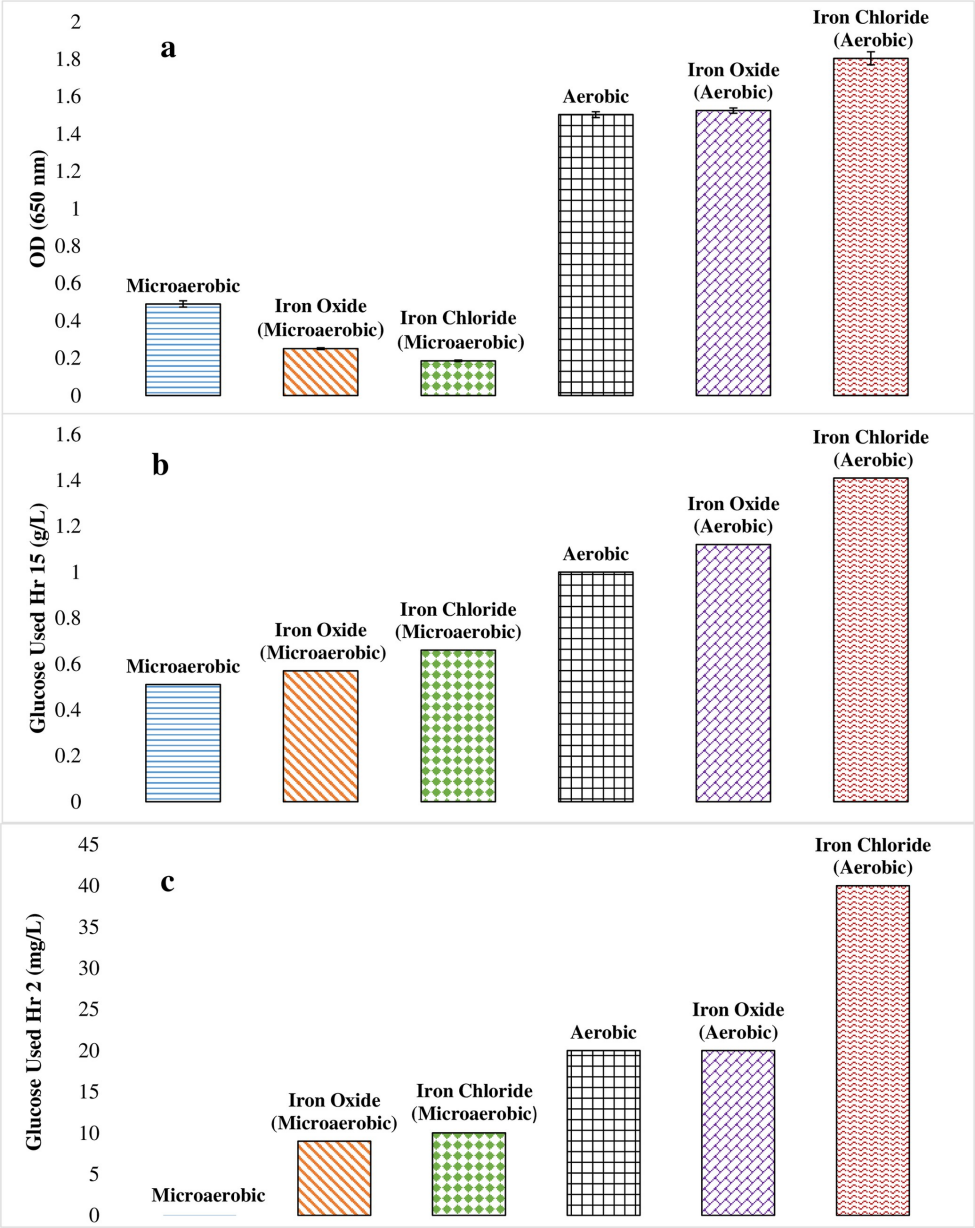
A comparison of a) *S*. *oneidensis* growth to b) the microbe’s utilization of glucose after 15 hours and c) after 2 hours of initial growth. Glucose usage increases with increasing oxygen concentration and upon the addition of ferric iron.

**Fig 5:**
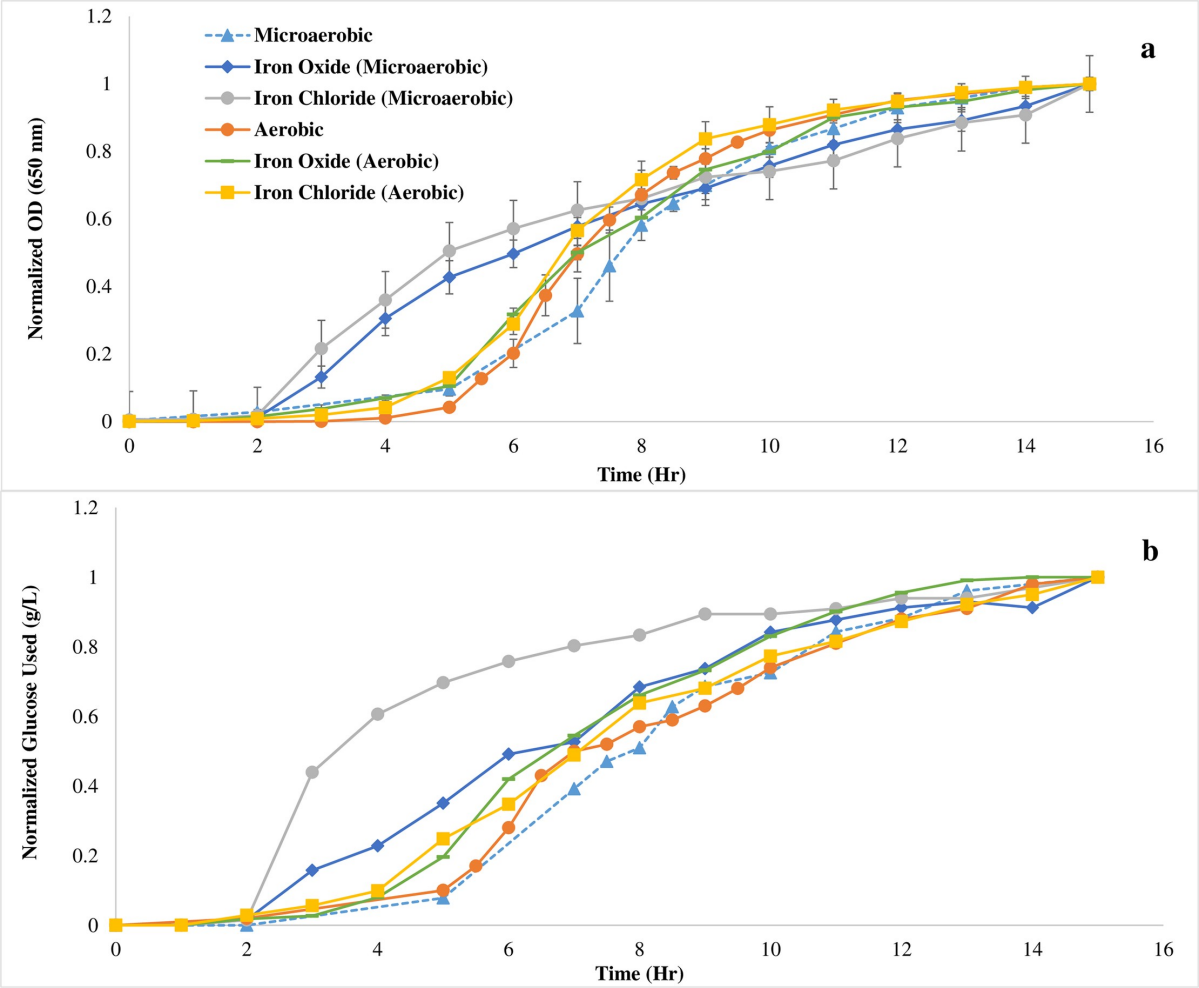
Normalized results for a) optical density and b) glucose usage. The onset of exponential growth appears sooner for microaerobic samples containing ferric iron in the growth media, which also correlates to the time at which glucose utilization becomes more rapid.

The applicability of the method was investigated through the quantitation of glucose consumed by *S*. *oneidensis* during early growth development. Cell cultures were grown for 15 hours. The turbidity of the cultures was measured at 30 to 60 minute intervals. The optical density achieved was dependent on the growth conditions (Fig 4A). The maximum growth was highest under aerobic compared to microaerobic conditions with oxygen rich cultures exhibiting 5 times the turbidity achieved by their oxygen deficient counterparts. The optical density disparity between the microaerobic and aerobic cultures correlates to the use of glucose by *S*. *oneidensis* (Fig 4B). Aerobic cultures utilized the most glucose after 15 hours. The addition of oxygen in the growth media resulted in a 2-fold increase in glucose utilization compared to microaerobic cultures. Microaerobic cultures containing Fe(III) as the predominate electron acceptor exhibited a more rapid onset of exponential growth compared to all other growth conditions (Fig 5A). These results agree with *S*. *oneidensis’s* ability to use various terminal electron acceptors to sustain growth [39–41]. Additionally, this early onset correlates to the time at which glucose begins to be utilized (Fig 5B).

The strength of the method is apparent when examining culture samples grown for two hours. Of the six culture types, the method could detect glucose across all but the microaerobic culture (Fig 4C). Measured glucose usage after five hours ranges between 9 to 40 mg/L. Comparable utilization times have not been reported in previous glucose studies [21–23]. Interestingly, the method revealed an ability of *S*. *oneidensis* to utilize a combination of electron acceptors to facilitate both microbial growth and consumption of glucose. These results agree with those proposed by Choi et al. [23] and Serres and Riley [32] indicating that *S*. *oneidensis* can grow in the presence of glucose only once oxygen is present within the media environment. When oxygen is limited, *S*. *oneidensis* exhibited stunted growth regardless of the presence of ferric iron. Further evidence for stunted growth comes when examining the two-hour growth cultures. Microaerobic cultures, except for those cultures containing iron chloride, did not register glucose usage suggesting the microbe has difficulty using glucose under these conditions. These results may indicate that oxygen is the preferred electron acceptor, and that the use of glucose when iron chloride is present may occur though a separate metabolism.

## Conclusions

An accurate and robust method was developed for analysis of glucose by derivatization with 1-phenyl-3-methyl-5-pyrazolone (PMP). The developed method focused on achieving low detection limits and reduced run times when compared to similar works. The applicability of the method was tested on microbial samples and was shown to be capable in determining the levels of glucose throughout the growth of *Shewanella oneidensis* MR-1. The developed method was sensitive and capable quantify glucose usage in microbial cultures after only two hours.

## Supporting information

S1 Fig Linearity of PMP-glucose with increasing concentration. The lower limit represents the LOQ (0.2 nM) while the upper limit corresponds to 120% of the expected glucose in TSB (3 g/L).
(TIF)

S1 Table Robustness study. (DOCX)

S2 Table Accuracy study. (DOCX)

S3 Table Precision studies. (DOCX)
