# Supplementary figures and images for: Development of a RP-HPLC method for determination of glucose in Shewanella oneidensis cultures utilizing 1-phenyl-3-methyl-5-pyrazolone derivatization
Source: PLoS One. 2020 Mar 12;15(3):e0229990. doi: 10.1371/journal.pone.0229990 (PMC7067395; doi:10.1371/journal.pone.0229990)

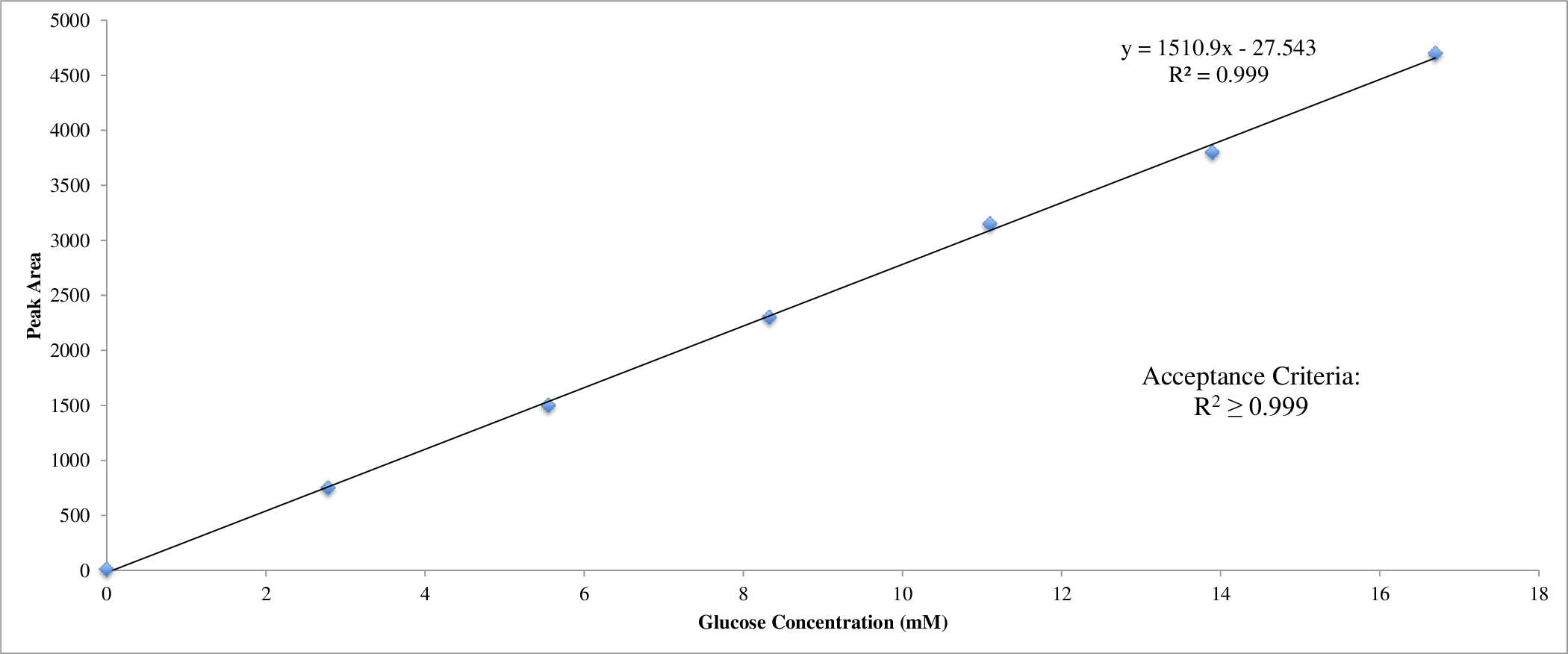

Supplement: S1 Fig — The lower limit represents the LOQ (0.2 nM) while the upper limit corresponds to 120% of the expected glucose in TSB (3 g/L). (TIF) [file pone.0229990.s001.tif]
