# Supplementary material for: Development of a RP-HPLC method for determination of glucose in Shewanella oneidensis cultures utilizing 1-phenyl-3-methyl-5-pyrazolone derivatization
Source: PLoS One. 2020 Mar 12;15(3):e0229990. doi: 10.1371/journal.pone.0229990 (PMC7067395; doi:10.1371/journal.pone.0229990)
Supplement: S1 Table — (DOCX) [file pone.0229990.s002.docx]

S1 Table: Robustness Study

| **Parameter** | **Condition** | **Resolution** | **Average LSL** |
| --- | --- | --- | --- |
| Wavelength (nm) | 247 | 5.80 | 6.03 |
|  | 245 | 6.15 |  |
|  | 243 | 6.13 |  |
| Flow Rate (mL/min) | 1.1 | 5.85 | 5.98 |
|  | 1.0 | 6.15 |  |
|  | 0.9 | 5.95 |  |
| Buffer pH | 7.4 | 6.17 | 6.11 |
|  | 7.2 | 6.15 |  |
|  | 7.0 | 6.02 |  |
| Ionic Strength (mM) | 17 | 6.13 | 6.13 |
|  | 15 | 6.15 |  |
|  | 13 | 6.10 |  |
| Temperature (°C) | 31.5 | 6.06 | 6.11 |
|  | 29.5 | 6.15 |  |
|  | 27.5 | 6.13 |  |
