# Supplementary material for: Development of a RP-HPLC method for determination of glucose in Shewanella oneidensis cultures utilizing 1-phenyl-3-methyl-5-pyrazolone derivatization
Source: PLoS One. 2020 Mar 12;15(3):e0229990. doi: 10.1371/journal.pone.0229990 (PMC7067395; doi:10.1371/journal.pone.0229990)
Supplement: S2 Table — (DOCX) [file pone.0229990.s003.docx]

S2 Table: Accuracy Study

| **Concentration (g/L)** | **Mean % Recovery (n=3)** |
| --- | --- |
| 2.5 | 100.5 ± 1.04 |
| 1.5 | 98.1 ± 0.665 |
| 0.5 | 101 ± 0.49 |
| Acceptance Criteria 98-102% | |
