# Supplementary material for: Development of a RP-HPLC method for determination of glucose in Shewanella oneidensis cultures utilizing 1-phenyl-3-methyl-5-pyrazolone derivatization
Source: PLoS One. 2020 Mar 12;15(3):e0229990. doi: 10.1371/journal.pone.0229990 (PMC7067395; doi:10.1371/journal.pone.0229990)
Supplement: S3 Table — (DOCX) [file pone.0229990.s004.docx]

S3 Table: Precision Studies

| **Test** | **Mean Area (n=6)** | **%RSD** |
| --- | --- | --- |
| Injection Precision (2.5 g/L) | 3865.57 ± 1.46 | 0.093 |
| Method Precision  (1.5 g/L) | 2230.3 ± 1.47 | 0.161 |
| Acceptance Criteria: %RSD ≤ 1.5% | | |
